# Sorafenib inhibits therapeutic induction of necroptosis in acute leukemia cells

**DOI:** 10.18632/oncotarget.19919

**Published:** 2017-08-04

**Authors:** Friederike Feldmann, Barbara Schenk, Sofie Martens, Peter Vandenabeele, Simone Fulda

**Affiliations:** ^1^ Institute for Experimental Cancer Research in Pediatrics, Goethe-University, Frankfurt, Germany; ^2^ German Cancer Consortium (DKTK), Partner Site, Frankfurt, Germany; ^3^ German Cancer Research Center (DKFZ), Heidelberg, Germany; ^4^ Inflammation Research Center, VIB, Ghent, Belgium; ^5^ Department of Biomedical Molecular Biology, Ghent University, Ghent, Belgium

**Keywords:** necroptosis, cell death, Sorafenib, Smac, leukemia

## Abstract

Induction of necroptosis has emerged as an alternative approach to trigger programmed cell death, in particular in apoptosis-resistant cancer cells. Recent evidence suggests that kinase inhibitors targeting oncogenic B-RAF can also affect Receptor-interacting serine/threonine-protein kinase (RIP)1 and RIP3. Sorafenib, a multi-targeting kinase inhibitor with activity against B-RAF, is used for the treatment of acute leukemia. In the present study, we therefore investigated whether Sorafenib interferes with therapeutic induction of necroptosis in acute leukemia. Here, we report that Sorafenib inhibits necroptotic signaling and cell death in two models of necroptosis in acute leukemia. Sorafenib significantly reduces Second mitochondria-derived activator of caspases (Smac) mimetic-induced necroptosis in apoptosis-resistant acute myeloid leukemia (AML) cells as well as Smac mimetic/Tumor Necrosis Factor (TNF)α-induced necroptosis in FADD-deficient acute lymphoblastic leukemia (ALL) cells. Sub- to low micromolar concentrations of Sorafenib corresponding to its plasma levels reported in cancer patients are sufficient to inhibit necroptosis, emphasizing the clinical relevance of our findings. Furthermore, Sorafenib blocks Smac mimetic-mediated phosphorylation of mixed-lineage kinase domain-like protein (MLKL) that marks its activation, indicating that Sorafenib targets components upstream of MLKL such as RIP1 and RIP3. Intriguingly, Sorafenib reduces the Smac mimetic/TNFα-stimulated interaction of RIP1 with RIP3 and MLKL, demonstrating that it interferes with the assembly of the necrosome complex. Importantly, Sorafenib significantly protects primary, patient-derived AML blasts from Smac mimetic-induced necroptosis. By demonstrating that Sorafenib limits the anti-leukemic activity of necroptosis-inducing drugs in acute leukemia cells, our study has important implications for the use of Sorafenib in the treatment of acute leukemia.

## INTRODUCTION

Necroptosis has recently been identified as a non-apoptotic form of programmed cell death and the serine/threonine kinases RIP1 and RIP3 constitute key elements of the necroptosis signaling machinery [1]. A prototypic signaling pathway to necroptosis is engaged by the binding of TNFα to its cognate cell surface receptor TNF receptor 1 (TNFR1), which triggers formation of the so-called complex I at the TNFR1 [1]. This leads to K63-linked polyubiquitination of RIP1 by cellular Inhibitor of Apoptosis (cIAP) proteins and activation of canonical Nuclear Factor kappaB (NF-κB). Upon internalization of TNFR1, secondary cell death complexes assemble in the cytosol. When caspase-8 activation is blocked, RIP1 is no longer cleaved and can interact with RIP3 to build up the necrosome resulting in activation of RIP1 and RIP3 in an autocrine/paracrine manner via reciprocal phosphorylation [2–4]. RIP3 subsequently phosphorylates and activates MLKL, a pseudokinase that lacks intrinsic kinase activity [5, 6]. Upon its activation, MLKL forms oligomers and translocates from the cytosol to the plasma and intracellular membranes, where it disrupts membrane integrity [7, 8].

Necroptosis may offer an alternative option to trigger programmed cell death in cancer cells, as many cancers have evolved mechanisms to evade cell death [9]. In apoptosis-resistant cancers therapeutic induction of necroptosis may be of particular interest, as activated caspase-8 has been shown to cleave and thus inactivate RIP1 [10]. This implies that apoptosis-resistant cancer cells are particularly susceptible to necroptosis.

Inhibitor of Apoptosis (IAP) proteins are a family of proteins that are involved in the regulation of programmed cell death [11]. For example, cIAP1 and cIAP2 restrain necroptosis by mediating the polyubiquitination of RIP1 via their E3 ligase activity. Small-molecule inhibitors of IAP proteins that resemble Smac, i.e. Smac mimetics, stimulate autoubiquitination and proteasomal degradation of cIAP proteins, thereby promoting the assembly of the necrosome leading to necroptosis under conditions when caspase activation is inhibited [9].

The kinase domain of human RIP1 and RIP3 shares high sequence similarity with the kinase domain of B-RAF [12]. In addition, structural comparison of inhibitor-bound RIP1 with the inhibitor-bound oncogenic kinase B-RAF revealed partially overlapping binding sites for the RIP1 inhibitor necrostatin-1 (Nec-1) and the B-RAF inhibitor Vemurafenib [12]. This suggests that kinase inhibitors that target oncogenic B-RAF might also affect RIP1 and/or RIP3. Indeed, the B-RAF inhibitor Dabrafenib has recently been shown to inhibit RIP3 independently of its effect on the B-RAF family members [13]. Sorafenib is a multi-targeting tyrosine kinase inhibitor with activity against B-RAF, but also against other kinases such as FLT3, VEGF, PDGF receptor and c-KIT [14]. It is used for the treatment of various malignancies including AML and ALL [15, 16]. In the present study, we therefore investigated the question as to whether or not Sorafenib interferes with necroptosis in apoptosis-resistant acute leukemia.

## RESULTS

### Sorafenib protects AML cells from BV6-induced necroptosis

**Figure 1:**
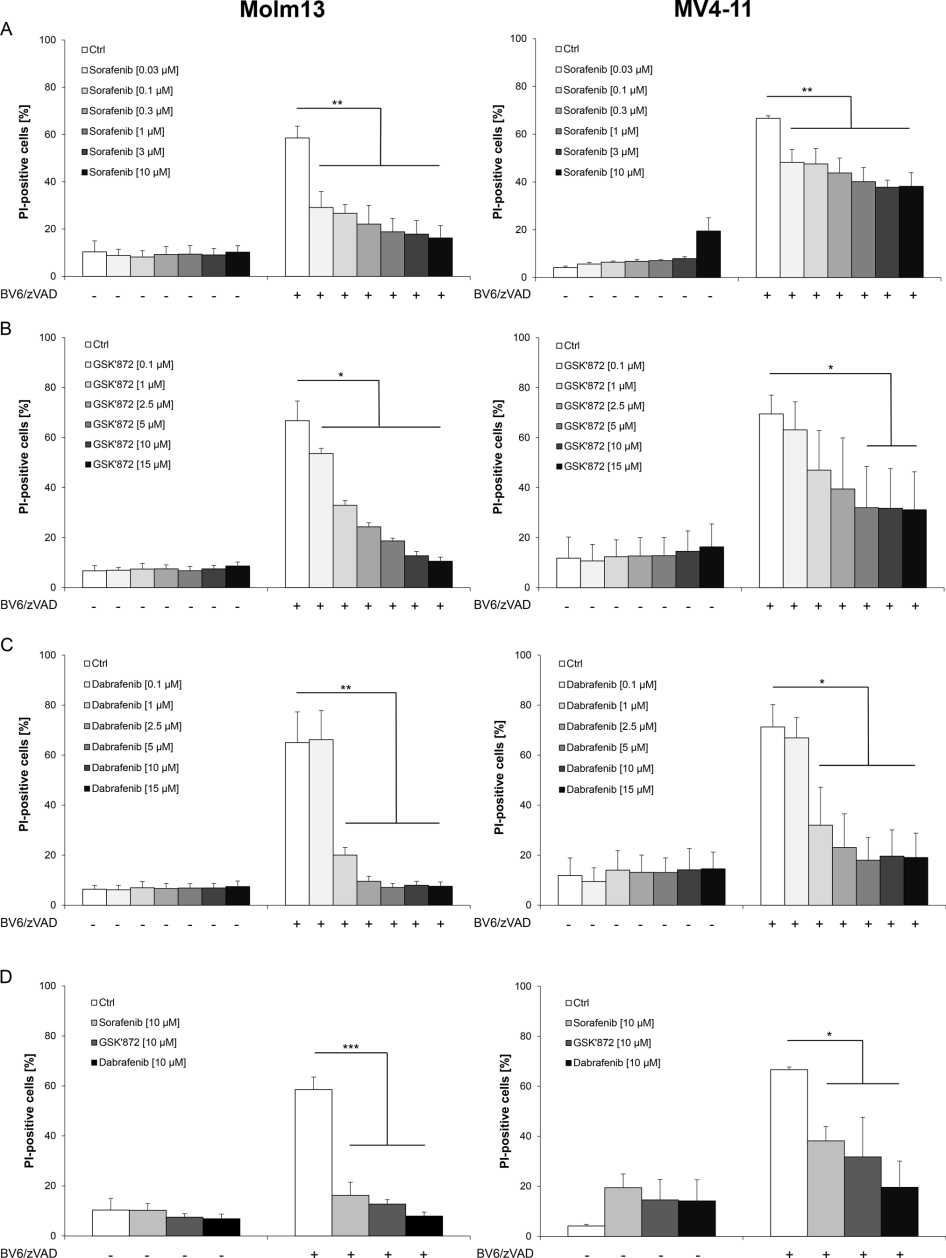

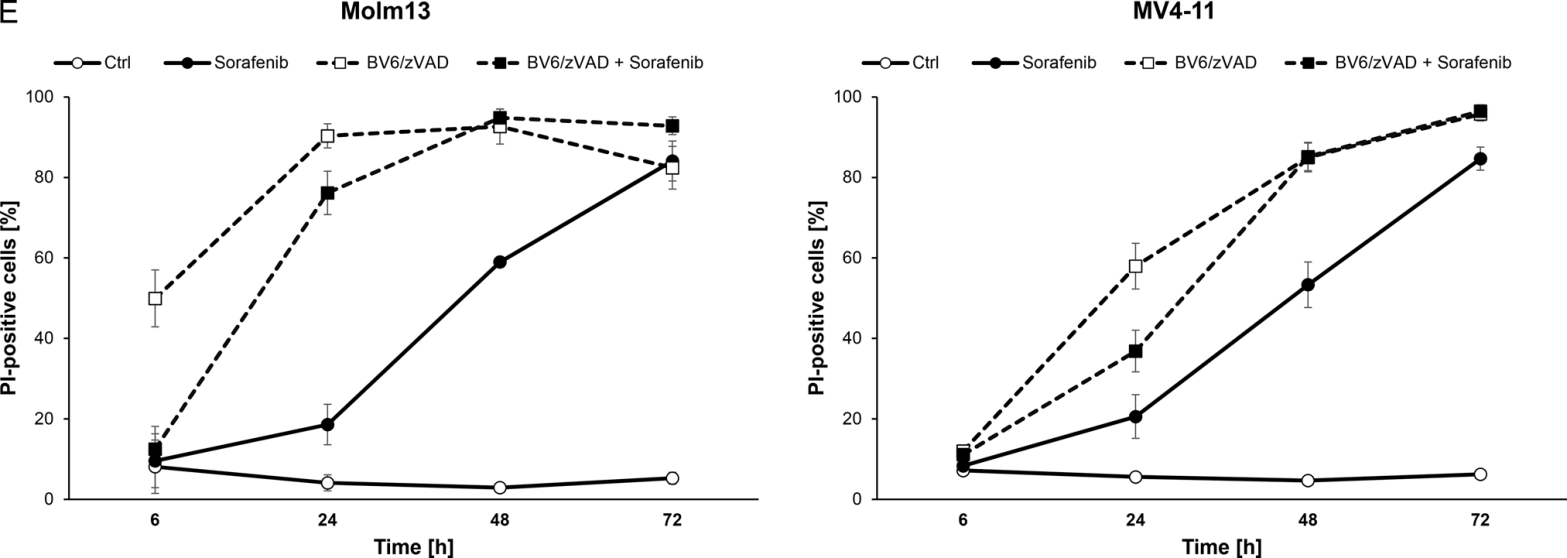
Sorafenib protects AML cells from BV6-induced necroptosis (**A**–**C**) Molm13 and MV4-11 cells were treated for 6 hours (Molm13) or 24 hours (MV4-11) with 3 μM BV6 and 40 μM zVAD.fmk in the presence or absence of indicated concentrations of Sorafenib (A), GSK’872 (B) or Dabrafenib (C). (**D**) Molm13 and MV4-11 cells were treated for 6 hours (Molm13) or 24 hours (MV4-11) with 3 μM BV6 and 40 μM zVAD.fmk in the presence or absence of 10 μM Sorafenib, 10 μM GSK’872 or 10 μM Dabrafenib. (**E**) Molm13 and MV4-11 cells were treated with or without 3 μM BV6 and 40 μM zVAD.fmk in the presence or absence of 10 μM Sorafenib for the indicated time points. (A–E) cell death was determined by PI staining and flow cytometry. Mean and SD of at least three experiments performed in triplicate are shown; *P < 0.05; **P < 0.01; ***P < 0.001.

We previously reported that Smac mimetics can engage necroptosis in AML cells expressing key components of necroptosis signaling when caspase activation and apoptosis are blocked [17]. To investigate whether Sorafenib modulates necroptosis in AML cells, we used the Smac mimetic BV6 in combination with the broad-range caspase inhibitor zVAD.fmk to trigger necroptotic cell death. Importantly, we found that the addition of Sorafenib at subtoxic concentrations significantly reduced BV6/zVAD.fmk-induced necroptosis in a dose-dependent manner in both Molm-13 and MV4-11 AML cells (Figure 1A).

To compare the anti-necroptotic activity of Sorafenib to an established RIP3 inhibitor, we used GSK’872, a small-molecule inhibitor described to block RIP3 [18]. GSK’872 significantly inhibited BV6/zVAD.fmk-induced cell death in AML cells (Figure 1B). Since the B-RAF inhibitor Dabrafenib has recently been reported to inhibit RIP3 by its adenosine-5′-triphosphate (ATP)-competitive binding to the enzyme [19], we also tested the effect of Dabrafenib on the induction of necroptosis in AML cells. Of note, the addition of Dabrafenib significantly reduced BV6/zVAD.fmk-stimulated cell death in Molm-13 and MV4-11 AML cells (Figure 1C). Comparison of equimolar concentrations of Sorafenib, Dabrafenib and GSK’872 in AML cells showed that Sorafenib and GSK’872 exhibited a comparable potency to reduce necroptotic cell death, while Dabrafenib turned out to be the most effective inhibitor in this model of necroptosis (Figure 1D).

Since exposure of AML cells to Sorafenib has been reported to elicit cell death [20, 21], we monitored cell death upon treatment with Sorafenib over an extended period of time. Indeed, Sorafenib induced cell death in AML cells in a time-dependent manner with a relatively slow kinetic and no or little cell death after six to 24 hours (Figure 1E). Furthermore, we investigated the long-term effect of Sorafenib on BV6/zVAD.fmk-induced necroptosis. A multi-day assay showed that Sorafenib protected both AML cell lines from BV6/zVAD.fmk-induced necroptosis for up to 24 hours, while it lost its protective effects later on, in parallel with becoming cytotoxic as single agent (Figure 1E). This shows that the inhibitory effect of Sorafenib on necroptosis is not only dose- but also time-dependent.

### Sorafenib inhibits BV6-induced phosphorylation of MLKL in AML cells

**Figure 2:**
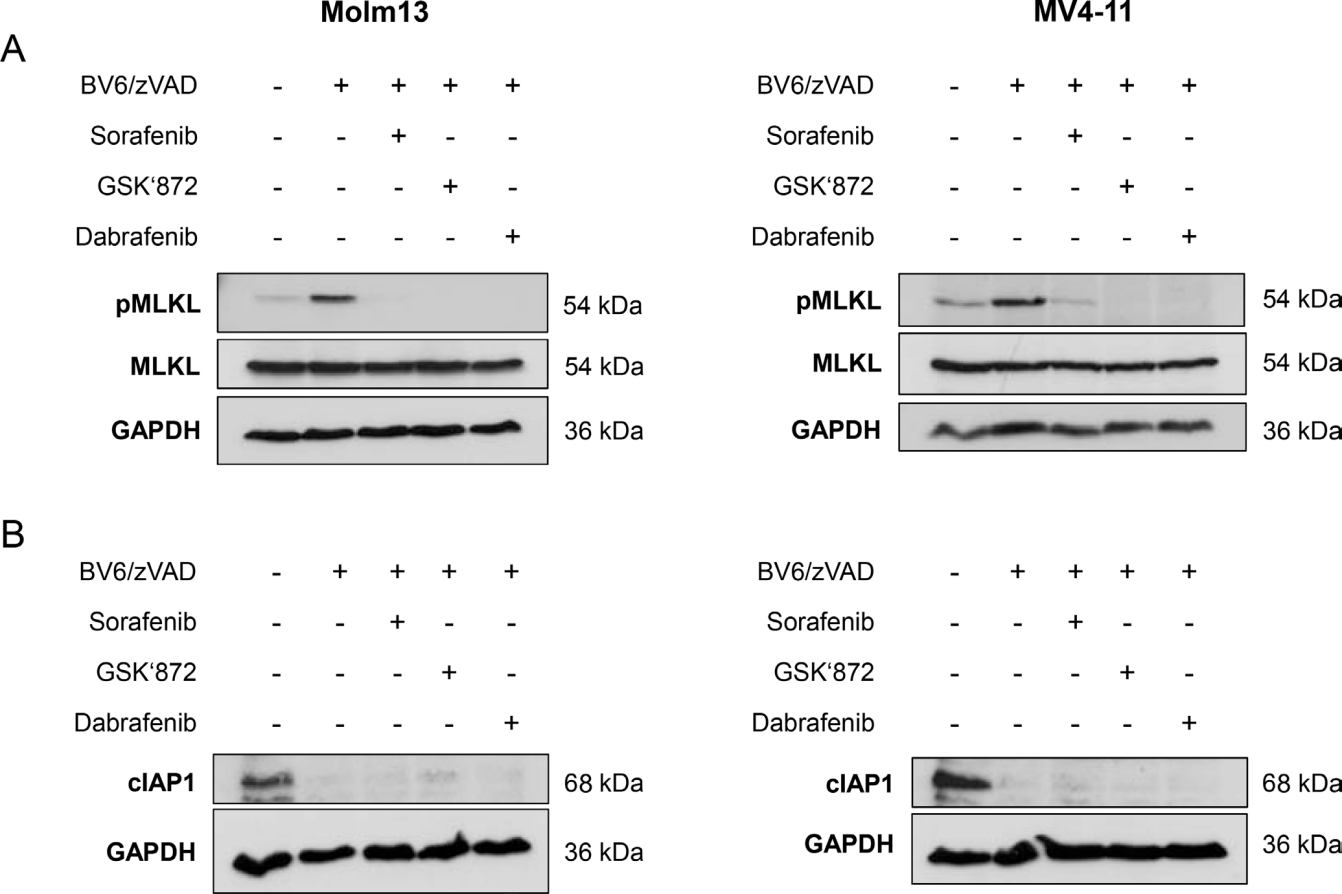
Sorafenib inhibits BV6-induced phosphorylation of MLKL in AML cells (**A**) Molm13 and MV4-11 cells were treated for 4½ hours (Molm13) or 11 hours (MV4-11) with 3 μM BV6 and 40 μM zVAD.fmk in the presence or absence of 10 μM Sorafenib, 15 μM GSK’872 or 5 μM Dabrafenib. (**B**) Molm13 and MV4-11 cells were treated for 5½ hours (Molm13) or 7 hours (MV4-11) with 3 μM BV6 and 40 μM zVAD.fmk in the presence or absence of 10 μM Sorafenib, 15 μM GSK’872 or 5 μM Dabrafenib. Expression levels of MLKL and phospho-MLKL (A) or cIAP1 (B) were assessed by Western blotting, GAPDH served as loading control.

Since MLKL is considered as a crucial mediator of necroptosis [1], we next investigated whether Sorafenib interferes with the phosphorylation of MLKL, which marks its activation [1]. Importantly, addition of Sorafenib almost completely prevented BV6/zVAD.fmk-stimulated phosphorylation of MLKL in both Molm-13 and MV4-11 AML cells (Figure 2A). Similarly, MLKL phosphorylation in response to treatment with BV6/zVAD.fmk was suppressed in the presence of GSK’872 or Dabrafenib (Figure 2A). This confirms that Sorafenib blocks BV6/zVAD.fmk-mediated necroptosis in AML cells and indicates that Sorafenib targets components upstream of MLKL, such as RIP1 and RIP3.

Smac mimetics act by neutralizing IAP proteins and binding of Smac mimetics to cIAP proteins causes their ubiquitination and proteasomal degradation [22, 23]. To confirm that Sorafenib inhibits necroptotic signaling rather than interfering with BV6-induced depletion of cIAP proteins, we assessed the effects of Sorafenib on expression levels of cIAP1 in response to BV6 treatment. cIAP2 protein levels were not assessed, as cIAP2 is not detectable in Molm-13 and MV4-11 AML cells [24]. Neither Sorafenib nor GSK’872 or Dabrafenib prevented the BV6-stimulated downregulation of cIAP1 (Figure 2B).

### Sorafenib protects ALL cells from BV6/TNFα-induced necroptosis

**Figure 3:**
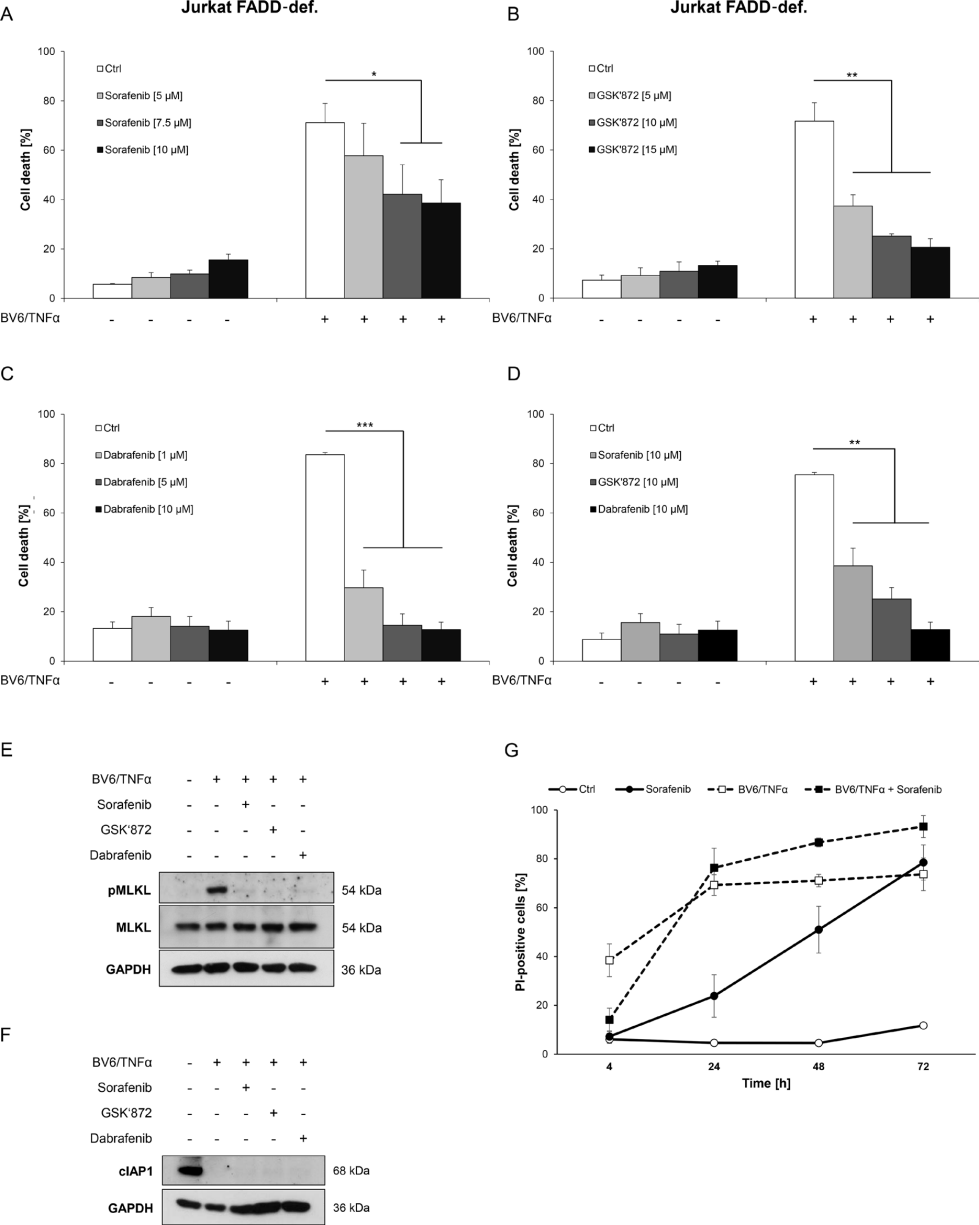
Sorafenib protects ALL cells from BV6/TNFα-induced necroptosis (**A**–**C**) FADD-deficient Jurkat cells were treated for 4 hours with 1 μM BV6 and 1 ng/ml TNFα in the presence or absence of indicated concentrations of Sorafenib (A), GSK’872 (B) or Dabrafenib (C). (**D**) FADD-deficient Jurkat cells were treated for 4 hours with 1 μM BV6 and 1 ng/ml TNFα in the presence or absence of 10 μM Sorafenib, 10 μM GSK’872 or 10 μM Dabrafenib. (**E** and **F**) FADD-deficient Jurkat cells were treated for 2 hours with 1 μM BV6 and 1 ng/ml TNFα in the presence or absence of 10 μM Sorafenib, 6 μM GSK’872 or 0.6 μM Dabrafenib. Expression levels of MLKL, phospho-MLKL and cIAP1 were analyzed by Western blotting, expression of GAPDH served as loading control. (**G**) FADD-deficient Jurkat cells were treated with or without 1 μM BV6 and 1 ng/ml TNFα in the presence or absence of 10 μM Sorafenib for the indicated time points. Cell death was determined by FSC/SSC analysis (A–D) or PI staining (G) and flow cytometry. Mean and SD of at least three experiments performed in triplicate are shown; *P < 0.05; **P < 0.01; ***P < 0.001.

We then extended our experiments to a necroptosis model of ALL, since Sorafenib is also used for the treatment of ALL [16]. To this end, we used FADD-deficient Jurkat T-cell ALL cells, which undergo necroptosis upon treatment with BV6 in combination with TNFα [25, 26]. Importantly, Sorafenib protected FADD-deficient Jurkat cells significantly at low micromolar concentrations from BV6/TNFα-induced necroptosis in a dose-dependent manner (Figure 3A). Similarly, BV6/TNFα-mediated cell death was significantly inhibited in the presence of GSK’872 or Dabrafenib (Figure 3B, 3C). Comparison of equimolar concentrations of these inhibitors demonstrated that Sorafenib is slightly less potent than GSK’872 to inhibit BV6/TNFα-induced necroptotic cell death in FADD-deficient Jurkat cells and that Dabrafenib is superior to the latter two inhibitors in this setting (Figure 3D). Furthermore, phosphorylation of MLKL upon treatment with BV6/TNFα was almost completely blocked in the presence of Sorafenib, GSK’872 or Dabrafenib in FADD-deficient Jurkat cells (Figure 3E). Similar to the AML model, neither Sorafenib nor the RIP3 inhibitors GSK’872 and Dabrafenib interfered with BV6-imposed depletion of cIAP1 (Figure 3F).

As Sorafenib has been reported to induce cell death in ALL cells [27], we monitored the effect of Sorafenib over time. Similar to the AML model, the kinetic of Sorafenib-triggered cell death was relatively slow with no or little cell death after six to 24 hours (Figure 3G). At an early time point Sorafenib inhibited BV6/TNFα-induced necroptosis in FADD-deficient Jurkat cells but not anymore after 24 hours of treatment, when it started to trigger cell death as single agent (Figure 3G). Together, this set of experiments shows that Sorafenib protects ALL cells from Smac mimetic/TNFα-induced necroptosis in a time-dependent fashion.

### Sorafenib inhibits BV6/TNFα-induced assembly of the necrosome in acute leukemia cells

**Figure 4:**
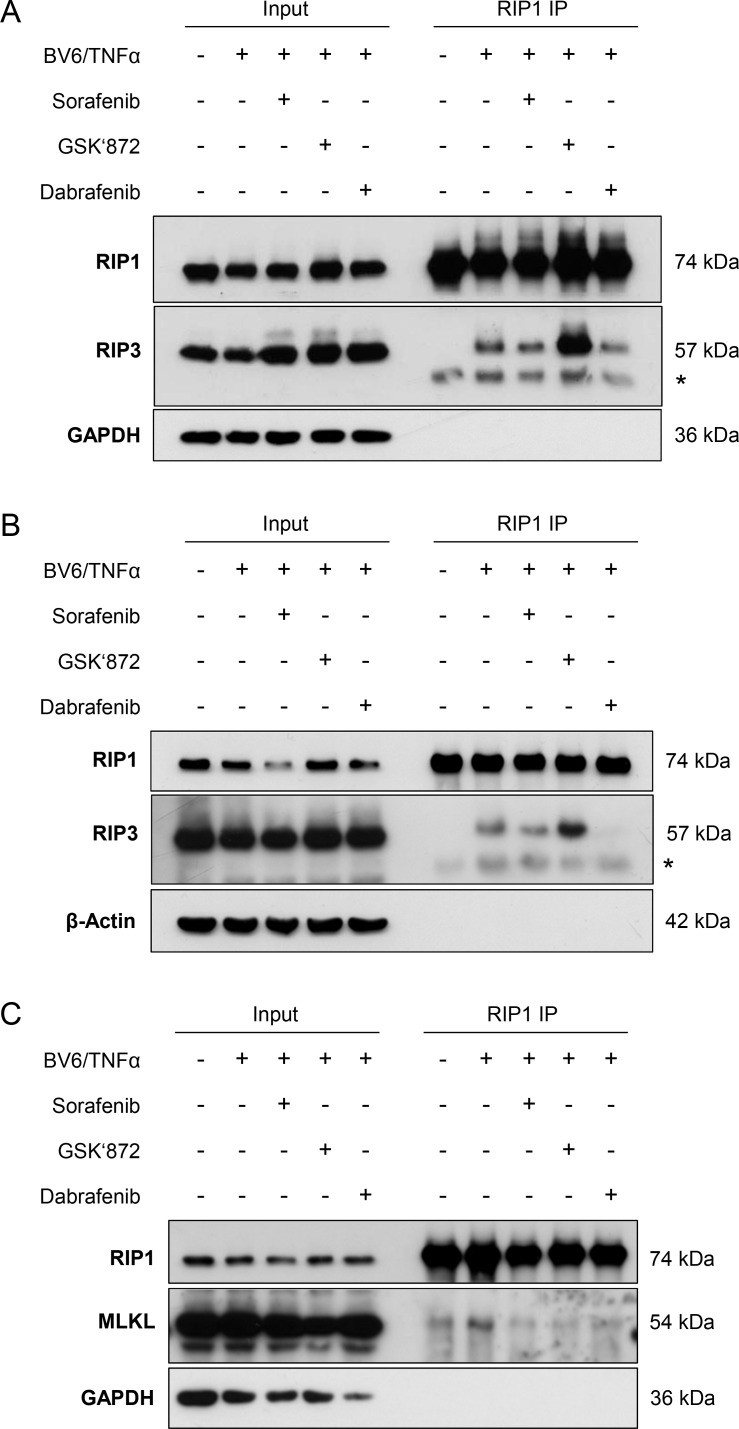
Sorafenib inhibits BV6/TNFα-induced assembly of the necrosome in acute leukemia cells (**A**) FADD-deficient Jurkat cells were treated for 2 hours with 1 μM BV6 and 1 ng/ml TNFα in the presence or absence of 10 μM Sorafenib, 6 μM GSK’872 or 0.6 μM Dabrafenib. RIP1 was immunoprecipitated using anti-RIP1 antibody and detection of indicated proteins was carried out by Western blotting; *: IgG heavy chain. (**B**) FADD-deficient Jurkat cells were treated for 2 hours with 1 μM BV6 and 1 ng/ml TNFα in the presence or absence of 10 μM Sorafenib, 10 μM GSK’872 or 5 μM Dabrafenib. RIP1 was immunoprecipitated using anti-RIP1 antibody and detection of indicated proteins was carried out by Western blotting; *: IgG heavy chain. (**C**) FADD-deficient Jurkat cells were treated for 2 hours with 1 μM BV6 and 1 ng/ml TNFα in the presence or absence of 10 μM Sorafenib, 6 μM GSK’872 or 0.6 μM Dabrafenib. RIP1 was immunoprecipitated using anti-RIP1 antibody crosslinked to beads and detection of indicated proteins was carried out by Western blotting.

As the necrosome complex represents a central signaling platform in necroptosis [1], we then asked whether Sorafenib alters the formation of the necrosome. To address this question, we treated FADD-deficient Jurkat cells with BV6/TNFα in the presence and absence of Sorafenib, immunoprecipitated RIP1 and analyzed its interaction with RIP3. Control experiments confirmed that we used equipotent concentrations of Sorafenib, GSK’872 and Dabrafenib for these immunoprecipitation studies that reduced BV6/TNFα-induced cell death to a comparable extent by about 50% (data not shown). Intriguingly, the presence of Sorafenib reduced the BV6/TNFα-stimulated interaction of RIP1 and RIP3 (Figure 4A). Similarly, Dabrafenib decreased binding of RIP3 to RIP1 in response to BV6/TNFα treatment (Figure 4A). We also observed that GSK’872 even promoted the interaction of RIP1 and RIP3 in BV6/TNFα-treated cells compared to cells that were stimulated with BV6/TNFα in the absence of GSK’872 (Figure 4A). A possible explanation for this finding is that GSK’872 alters the conformation of RIP3 thereby unleashing its RHIM-dependent oligomerization with RIP1 as recently reported [28].

To explore whether the ability of Dabrafenib to prevent necrosome assembly correlated with its ability to inhibit BV6/TNFα-stimulated necroptosis, we also tested a higher concentration of Dabrafenib. Of note, Dabrafenib at a concentration of 5 μM, which entirely abrogated BV6/TNFα-stimulated cell death (Figure 3C), almost completely abolished the interaction of RIP1 and RIP3 in response to BV6/TNFα treatment (Figure 4B).

Furthermore, we investigated the question as to whether or not Sorafenib regulates the binding of MLKL to the necrosome complex. To this end, we immunoprecipitated RIP1 and analyzed its interaction with MLKL. Interestingly, the presence of Sorafenib reduced the BV6/TNFα-stimulated interaction of RIP1 and MLKL (Figure 4C). Similarly, the interaction of RIP1 and MLKL upon BV6/TNFα treatment was attenuated in the presence of GSK’872 or Dabrafenib (Figure 4C). This set of experiments demonstrates that Sorafenib interferes with the assembly of the necrosome during BV6/TNFα-induced necroptosis in leukemia cells.

### Sorafenib does not inhibit Smac mimetic-induced apoptosis in acute leukemia cells

**Figure 5:**
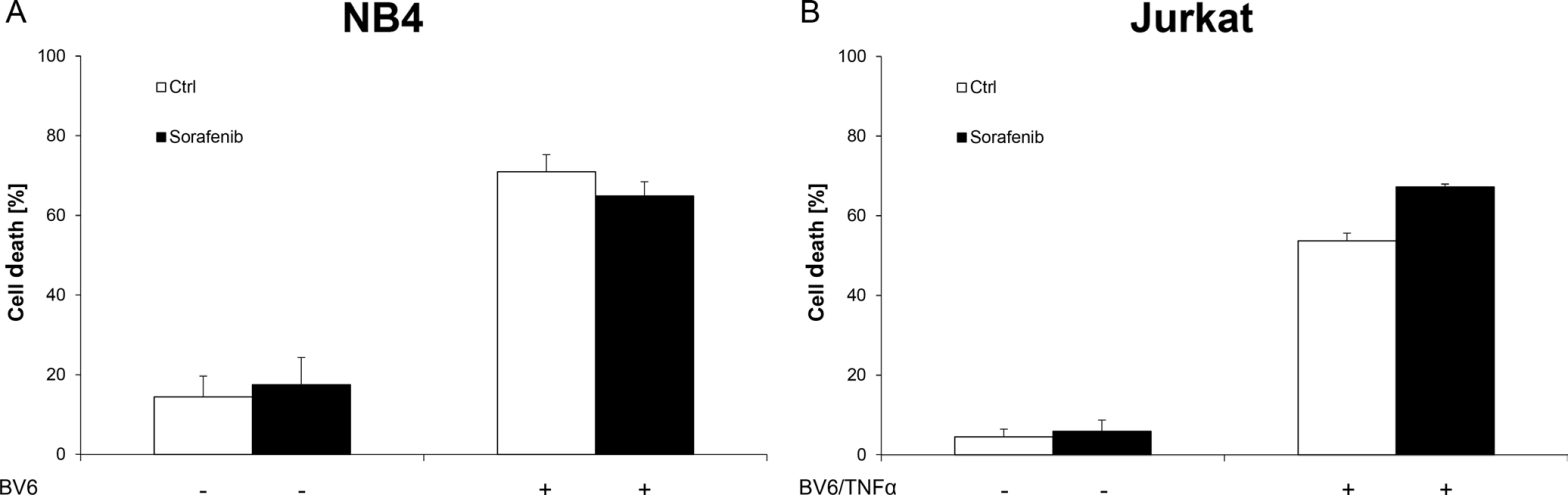
Sorafenib does not inhibit BV6-induced apoptosis in acute leukemia cells (**A**) NB4 cells were treated for 7 hours with 7 μM BV6 in the presence or absence of 10 μM Sorafenib. (**B**) Jurkat cells were treated for 24 hours with 5 μM BV6 and 1 ng/ml TNFα in the presence or absence of 5 μM Sorafenib. Cell death was determined by FSC/SSC analysis and flow cytometry. Mean and SD of at least three experiments performed in triplicate are shown; *P < 0.05; **P < 0.01; ***P < 0.001.

Next, we tested whether Sorafenib specifically inhibits BV6-induced necroptosis in acute leukemia cells. To this end, we investigated the effect of Sorafenib on BV6-induced apoptosis in RIP3-deficient and therefore necroptosis-resistant NB4 AML cells as well as in Jurkat wild-type cells, which have previously been shown to undergo apoptosis upon BV6/TNFα treatment that is blocked by the caspase inhibitor zVAD.fmk [17, 26]. Of note, Sorafenib failed to rescue NB4 and Jurkat cells from BV6-induced apoptosis (Figure 5A, 5B).

These findings demonstrate that Sorafenib does not protect acute leukemia cells from Smac mimetic-induced apoptosis.

### Sorafenib rescues primary AML cells from BV6-induced necroptosis

**Figure 6:**
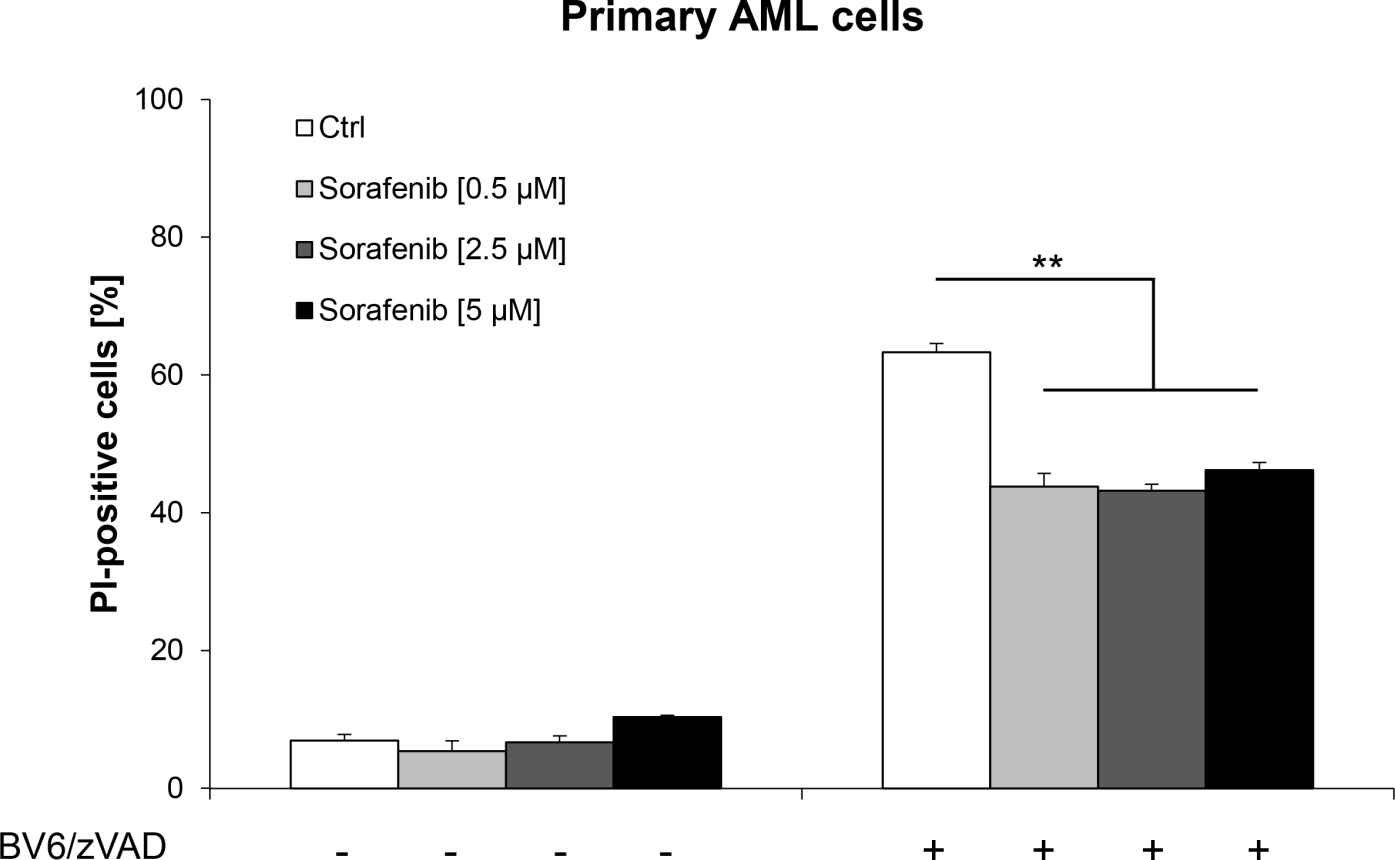
Sorafenib rescues primary AML cells from BV6-induced necroptosis Primary AML cells were treated for 36 hours with 10 nM BV6 and 20 μM zVAD.fmk in the presence or absence of indicated concentrations of Sorafenib. Cell death was determined by PI staining and flow cytometry. Mean and SD of triplicates are shown; *P < 0.05; **P < 0.01; ***P < 0.001.

To investigate the clinical relevance of our results, we studied the effects of Sorafenib on necroptosis induction in primary leukemic blasts freshly isolated from a patient with AML at diagnosis. Importantly, non-toxic concentrations of Sorafenib substantially reduced BV6/zVAD.fmk-induced cell death in primary AML cells (Figure 6). This demonstrates that Sorafenib impairs Smac mimetic-induced necroptosis not only in AML cell lines but also in primary, patient-derived AML blasts.

## DISCUSSION

Sorafenib is a multi-targeting tyrosine kinase inhibitor that is used for the treatment of e.g. acute leukemia [15, 16]. In the present study, we investigated whether Sorafenib interferes with therapeutic induction of necroptosis in acute leukemia.

Here, we report that Sorafenib inhibits necroptotic signaling and cell death in acute leukemia cells, when caspase activation and apoptosis are blocked by FADD deficiency or zVAD.fmk. This conclusion is supported by a number of arguments. First, Sorafenib inhibits necroptosis at sub- to low micromolar concentrations with a comparable potency to the small-molecule inhibitor GSK’872 that has been described to block RIP3 [18]. Second, Sorafenib interferes with the assembly of the necrosome complex upon the induction of necroptosis reducing the interaction of RIP1 with RIP3 and MLKL. Third, Sorafenib strongly attenuates phosphorylation of MLKL, a posttranslational modification that is mediated by RIP3 and marks its activation [5]. This indicates that Sorafenib targets components upstream of MLKL such as RIP1 and RIP3.

Sorafenib is a multi-targeting kinase inhibitor that inhibits B-RAF in addition to other kinases [14]. The fact that it inhibits RIP1 and RIP3 in addition to its known targets is supported by recent structural biology studies showing a strong structural homology between the kinase domains of both human RIP1 and RIP3 and the kinase domain of B-RAF [12]. This underscores that kinase inhibitors targeting oncogenic B-RAF might also affect RIP1 and RIP3. Indeed, there is recent evidence indicating that Sorafenib inhibits RIP1 and RIP3 kinase activity [29]. Sorafenib has also been reported to induce necroptosis based on data showing that the RIP1 inhibitor Nec-1 attenuated Sorafenib-mediated cell death in multiple myeloma, Hodgkin lymphoma or prostate carcinoma cells [30–32]. However, as RIP1 can control necroptotic as well as apoptotic pathways, Nec-1-conferred protection from cell death cannot be taken as sufficient evidence for necroptosis.

Besides Sorafenib, other kinase inhibitors that are currently being used as anticancer drugs have been reported to exhibit anti-necroptotic effects. The B-RAF inhibitor Dabrafenib has been shown to inhibit RIP3 through ATP-competitive binding to the enzyme, to inhibit necroptosis in cellular models and to alleviate toxic liver injury *in vivo* [13]. This Dabrafenib-imposed protection from necroptosis was described to occur independently of its effect on the B-RAF family members [13]. Consistently, we show in the present study that Dabrafenib protects acute leukemia cells from therapeutic induction of necroptosis. Furthermore, a cellular screen with Food and Drug Administration (FDA)-approved drugs identified the structurally distinct multi-targeting kinase inhibitors Pazopanib and Ponatinib as necroptosis inhibitors [33]. Ponatinib is a multikinase inhibitor, which targets BCR-ABL besides other kinases such as VEGF receptor, PDGF receptor, FGFR, FLT3 and c-KIT [34] and has been developed for the treatment of Philadelphia chromosome-positive acute leukemia [35]. Ponatinib has been shown to inhibit both RIP1 and RIP3 via direct binding [33, 36]. The protective effect of Pazopanib, a receptor tyrosine kinase inhibitor targeting VEGF receptors, PDGF receptor and c-KIT [37], has been reported to be mediated via RIP1 as the main functional target [33]. Together, these reports show that several multi-kinase inhibitors with different activity spectra can protect from necroptosis by targeting RIP1 and/or RIP3.

The data on hand showing that Sorafenib can limit the anti-leukemic activity of necroptosis-inducing drugs in acute leukemia cells has important implications, as Sorafenib is currently being used in the clinic for the treatment of AML and ALL [15, 16]. The induction of necroptosis has emerged in recent years as an alternative therapeutic approach to trigger programmed cell death in cancer cells, in particular in apoptosis-resistant cases [38]. In AML, we recently demonstrated that Smac mimetics alone or in combination with standard chemotherapeutic drugs such as cytarabine or epigenetic modifiers (i.e. demethylating agents, histone deacetylase (HDAC) inhibitors) can overcome apoptosis resistance of AML cells by inducing necroptosis as an alternative mode of programmed cell death [17, 24, 39, 40]. Also, Smac mimetics together with glucocorticoids, demethylating agents or TNFα have been shown to elicit necroptosis in apoptosis-resistant ALL cells [25, 41, 42]. Our current study demonstrating that Sorafenib at sub- to low micromolar concentrations inhibits treatment-induced necroptosis in AML and ALL cells implies that Sorafenib may limit the anti-leukemic activity of anticancer drugs that trigger necroptosis under certain conditions, for example when caspase activation is blocked. Nevertheless, additional studies, e.g. using *in vivo* leukemia mouse models, are still outstanding to test whether Sorafenib may limit the antileukemic activity of necroptosis-inducing agents. Also, downregulation of RIP3 has been reported in some human cancers including AML [43, 44]. The clinical relevance of our findings is underscored by our data showing that Sorafenib impairs the therapeutic induction of necroptotic cell death by Smac mimetic not only in AML and ALL cell lines but also in primary, patient-derived AML blasts. Furthermore, the concentrations of Sorafenib exhibiting anti-necroptotic effects in leukemia cells correspond to plasma levels of 3–17 μM Sorafenib that have been reported in cancer patients upon treatment with Sorafenib [45, 46]. Taken together, our study has important implications for the use of Sorafenib in the treatment of acute leukemia.

## MATERIALS AND METHODS

### Cell culture and chemicals

AML cell lines were obtained from DSMZ (Braunschweig, Germany), FADD-deficient Jurkat cells were kindly provided by Dr. J. Blenis [47]. Cells were cultured in RPMI 1640 medium (Life Technologies, Inc., Eggenstein, Germany), supplemented with 10% or 20% fetal calf serum (FCS) (Biochrom, Berlin, Germany), 1% penicillin/streptomycin (Invitrogen, Karlsruhe, Germany) and 1 mM sodium pyruvate (Invitrogen) for AML cell lines or 2.5% HEPES (Invitrogen) for ALL cell lines. Primary AML cells were maintained in Iscove's Modified Dulbecco's Medium with *GlutaMAX™* supplement (Life Technologies) supplemented with 10% FCS, 1% penicillin/streptomycin, 1% sodium pyruvate, 1% non-essential amino acid, 8 ng/ml interleukin-3, 20 ng/ml granulocyte-macrophage colony-stimulating factor, 20 ng/ml stem cell factor and 50 μM 2-mercaptoethanol. Biomaterial and clinical data were obtained from the hematological biobank and the tumor documentation of the UCT Frankfurt (Germany), characteristics of the sample are summarized in Supplementary Table 1. The study protocol has been approved by the local ethics committee of the University Hospital Frankfurt and informed consent has been obtained. The Smac mimetic BV6, which neutralizes x-linked IAP (XIAP), cIAP1 and cIAP2 [22], was kindly provided by Genentech, Inc. (South San Francisco, CA, USA). Caspase inhibitor zVAD.fmk was obtained from Bachem (Heidelberg, Germany), TNFα from Biomol (Hamburg, Germany), GSK’872 from Merck (Darmstadt, Germany), Sorafenib from Bayer AG (Leverkusen, Germany), Dabrafenib from Selleckchem (Houston, TX, USA). All chemicals were purchased by Sigma-Aldrich (Taufkirchen, Germany) or Carl Roth (Karlsruhe, Germany) unless indicated otherwise. Cells were preincubated with inhibitors (zVAD.fmk, Sorafenib, GSK’872 or Dabrafenib) for one hour before treatment. BV6 was applied two hours before stimulation with TNFα.

### Western blot analysis

Western blot analysis was performed as described previously [48] using the following antibodies: cIAP1 (R&D Systems, Inc., Wiesbaden, Germany), RIP1 (BD Biosciences, Heidelberg, Germany), RIP3 (Novus Biologicals, Littleton, CO, USA), MLKL (GeneTex, Irvine, CA, USA), phospho-MLKL (Cell Signaling Technologies, Danvers, MA, USA), β-Actin (Sigma-Aldrich) and glyceraldehyde 3-phosphate dehydrogenase (GAPDH) (HyTest, Turku, Finland) as loading controls and secondary antibodies conjugated to horseradish peroxidase (Santa Cruz Biotechnology, Santa Cruz, CA, USA). Enhanced chemiluminescence was used for detection (Amersham Bioscience, Freiburg, Germany). Alternatively, secondary antibodies labeled with IRDye infrared dyes were used for fluorescence detection (Odyssey Imaging System, LI-COR Bioscience, Bad Homburg, Germany). All Western blots shown are representative of at least two independent experiments.

### Determination of cell death

Cell death was assessed by forward/side scatter (FSC/SSC) analysis and flow cytometry as described previously [49] or by determination of plasma membrane permeability using propidium iodide (PI) staining (Sigma-Aldrich) and flow cytometry.

### Immunoprecipitation

RIP1 immunoprecipitation was performed as previously described [25]. Briefly, cells were lysed in nonidet P-40 (NP-40) buffer (10 mM Tris (pH 8.0), 150 mM NaCl, 1% NP-40), supplemented with protease inhibitor cocktail (Roche, Grenzach, Germany) and phosphatase inhibitors (1 mM sodium orthovanadate, 1 mM β-glycerophosphate, 5 mM sodium fluoride). At least 1.5 mg of protein was incubated with 1.5 μg of mouse anti-RIP1 antibody (no. 610459; BD Biosciences) overnight at 4°C followed by the addition of 15 μl pan-mouse IgG Dynabeads (Invitrogen), and then rotated for at least two hours at 4°C and washed with NP-40 buffer. Denaturated samples were analyzed by Western blot for the expression of RIP1, RIP3 and MLKL. For analyzing the interaction of RIP1 and MLKL, the RIP1 antibody was crosslinked to beads using dimethyl pimelimidate ((DMP); Thermo Scientific, Rockford, USA).

### Statistical analysis

Statistical significance was assessed by Student's *t*-test (two-tailed distribution, two-sample, unequal variance).

## SUPPLEMENTARY MATERIALS FIGURES AND TABLES



## Abbreviations

ALL: acute lymphoblastic leukemia
AML: acute myeloid leukemia
ATP: adenosine-5′-triphosphate
cIAP: cellular Inhibitor of Apoptosis
DMP: dimethyl pimelimidate
FADD: Fas-Associated protein with Death Domain
FCS: fetal calf serum
FDA: Food and Drug Administration
FSC/SSC: forward/side scatter
GAPDH: glyceraldehyde 3-phosphate dehydrogenase
HDAC: histone deacetylase
IAP: Inhibitor of Apoptosis
MLKL: mixed-lineage kinase domain-like protein
Nec-1: necrostatin-1
NF-κB: Nuclear Factor kappaB
PI: propidium iodide
RIPK: receptor-interacting serine/threonine-protein kinase
Smac: Second mitochondria-derived activator of caspases
TNF: Tumor Necrosis Factor
TNFR: TNF receptor
XIAP: x-linked IAP
