# Supplementary material for: Sorafenib inhibits therapeutic induction of necroptosis in acute leukemia cells
Source: Oncotarget. 2017 Aug 4;8(40):68208–20. doi: 10.18632/oncotarget.19919 (PMC5620249; doi:10.18632/oncotarget.19919)
Supplement: Supplementary file 1 [file oncotarget-08-68208-s001.pdf]

Sorafenib inhibits therapeutic induction of necroptosis in acute leukemia cells

SUPPLEMENTARY MATERIALS

Supplementary Table 1: Characteristics of primary AML sample

Patient	Type of material	Subtype [FAB]	Subtype [WHO]	Age	Sex	Karyotype	Genetic alteration
1	BM	M4	C92.0 Acute myeloid leukemia	74	Male	46, XY	NPM-1 mutation, FLT3-ITD

BM, bone marrow; FAB, criteria of the French-American-British (FAB) group; WHO, criteria of the World Health Organization.
